# Effects of corn oil ingestion on the intestinal mucosa of normal dogs

**DOI:** 10.3389/fvets.2024.1440942

**Published:** 2024-12-13

**Authors:** Su-Jin An, Young Joo Kim, Il-Hwa Hong, Dong-In Jung

**Affiliations:** ^1^Institute of Animal Medicine, College of Veterinary Medicine, Gyeongsang National University, Jinju, Republic of Korea; ^2^College of Veterinary Medicine, Western University of Health Sciences, Pomona, CA, United States

**Keywords:** capsule endoscopy, dog, endoscopy, enteropathy, lymphangiectasia, oil

## Abstract

**Introduction:**

We assessed corn oil’s oral effectiveness in detecting small bowel changes in healthy dogs through ultrasonography, endoscopy, and histopathology. We hypothesize that corn oil ingestion will not significantly increase the visibility of lymphatics and lacteals in healthy dogs.

**Methods:**

Five healthy male beagles were studied under institutional guidelines. The small intestine’s mucosal changes were observed post corn oil consumption (0.5 mL/kg) at various time intervals using ultrasonography, endoscopy, and histopathology. Ultrasonography was employed in real-time, and mucosal echogenicity scores were assigned at multiple time points. Endoscopic and capsule endoscopic examinations were conducted weekly at different intervals post oil consumption, with biopsy samples taken from the duodenal and ileal mucosa for histopathological evaluations.

**Results:**

Ultrasonographic evaluations showed no pathological conditions in any dog. While conventional endoscopic evaluations reflected normal variation, capsule endoscopy revealed significant duodenal and jejunal mucosal changes 3 h post-ingestion, but not in the ileum. Histopathological evaluation indicated a transient rise in the dilation of ileum villi 3 h post-ingestion, reducing by 12 h.

**Conclusion:**

In conclusion, this study demonstrated that the observed physiological changes in the small intestinal mucosa, including lymphatic dilation, hyperechoic speckles, and stripes, were within the normal range after oil ingestion in healthy Beagle dogs.

## 1 Introduction

The lymphatic system plays a crucial role in the absorption of nutrients and fluids from the gastrointestinal (GI) tract. Lacteals, located in the villi of the small intestine, transport lipid-rich filtrates into lymphatic vessels. Impairment of lymphatic function can lead to systemic consequences and affect lipid metabolism and transport (1). Protein-losing enteropathies (PLE) are characterized by the gradual loss of intestinal proteins due to elevated lymphatic pressure, lymphatic congestion, and mucosal diseases (2, 3).

In human medicine, inflammatory bowel diseases (IBD), such as Crohn’s disease and ulcerative colitis, are commonly associated with lymphatic dysfunction (2). Increased lymphangiogenesis, which may compensate for lymphatic insufficiencies, is observed during inflammation (2). In veterinary medicine, intestinal lymphangiectasia (IL) is one of the common forms of PLE in dogs and is characterized by dilation of the intestinal lymphatics (3). Although endoscopy is useful for diagnosing IL, the pathophysiology of lymphatic function and GI microcirculation remains poorly studied.

The high-fat diet challenge, which involves the oral administration of fat, has been proposed as a diagnostic tool to assess lymphatic flow and mucosal patterns in the small intestine. Different studies have utilized various methods and fat sources, but the optimal protocol has not been established. Some studies have suggested that a high-fat meal should be ingested just before the examination (4, 5), while others recommend ingestion the night before (6, 7). Fat intake stimulates enteric lymphatic flow and can be observed on ultrasound as bright speckles and striations (6, 8). While previous studies have suggested that feeding a high-fat diet prior to examination may enhance the diagnostic yield in dogs with lymphangiectasia, there is a lack of definitive research exploring the specific impact of high-dose long-chain triglyceride (LCT) oil on the small intestinal mucosa of normal dogs.

This study aims to evaluate the efficacy of orally administered corn oil as a high-fat meal in enhancing the detection of small bowel changes through ultrasonography, endoscopy, and histopathology in healthy dogs. We hypothesize that corn oil ingestion will not significantly increase the visibility of lymphatics and lacteals in healthy dogs.

## 2 Materials and methods

### 2.1 Animal preparation

Five healthy male beagles were selected for the study and treated in accordance with institutional guidelines (IACUC approval no. GNU-181001-D0052). The dogs exhibited no signs of gastrointestinal issues and underwent various tests, including blood work, urinalysis, fecal examination, and medical imaging, all of which showed normal results. The dogs weighed between 8.8 kg and 10.7 kg (mean weight, 9.75 kg) and fasted for 15 h before ultrasonographic examination and endoscopy. They were fed twice daily with Natural Balance’s Limited Ingredient Diets Potato & Duck Formula for 6 months with the amount given determined by their body weight. This commercial diet meets the nutritional requirements established by AAFCO for adult maintenance. The nutrient composition of the dog food in 2018 was 12.2% fat, 21% protein, and 46% carbohydrates on a dry matter basis. The daily maintenance demand energy requirement was calculated as 1.8 × resting energy requirement (70 × [body weight] 3/4). To adhere to the recommended fat intake, corn oil, long-chain triglycerides (LCTs) was used as a source of fat, with a dose of 0.5 mL per body weight deemed as appropriate. The materials and methods involved the selection and preparation of animals, including health assessments, fasting, diet, and fat intake considerations.

### 2.2 Ultrasonography

#### 2.2.1 Ultrasonographic instrument and standard procedure

Ultrasound studies were conducted using an ultrasound scanner (Arietta 70, Hitachi Aloka Medical, Japan) equipped with a high-frequency (12 MHz) linear-array transducer. The dogs were positioned in dorsal recumbency, and the ventral abdominal hair was clipped. Acoustic coupling gel was applied to the skin for optimal imaging. The dogs were awake and manually restrained during the examination.

The examination started by tracing the duodenum along its length on the right lateral abdomen, followed by tracing the jejunal segments in the left and right abdomen in a cranial to caudal direction. Scanning continued until the ileocecal valve was identified. Longitudinal and transverse images were obtained with medium or long internal focus and technical settings adjusted for optimal image quality. The entire small intestine was scanned in real time, and mucosal echogenicity scores were assigned at different time points: fasting, immediate (0 h), 1 h, 3 h, 6 h, and 12 h after the ingestion of 0.5 mL/kg of corn oil. The images were compared at different times to evaluate changes in sonographic interpretation based on the anatomical distinction of small intestine sections. The duodenum and ileum were identified based on their adjacent locations to the pylorus and ileocolic valve, respectively. The remaining mid-section images were considered representative of the jejunum. The presence or absence of specific mucosal echogenicity changes, such as hyperechoic mucosa, bright speckles, hyperechoic lines parallel to the submucosa, and perpendicular linear striations, were recorded for each time point and each dog. The findings were stored as DICOM images.

#### 2.2.2 Criteria for ultrasonographic evaluation of small intestine mucosa

A standard abdominal ultrasound examination was performed using general intestinal evaluation criteria, including small intestinal wall layering, movement via intestinal peristalsis, small intestinal wall thickness, small intestinal mucosal echogenicity, and mesenteric echogenicity.

##### 2.2.2.1 General ultrasonographic findings of small intestine mucosa

The overall structural integrity of wall layering was assessed, and the presence or absence of wall layers was recorded. Intestinal wall evaluations were performed on frozen images with the small intestinal segment in a long-axis orientation. The motility of the small intestine was evaluated, and the luminal diameter was measured to detect stenosis or dilatation. Wall thickness measurements were performed at several sites in the same section of the small bowel, with the thickest point selected as the measuring point. If the thickness of the bowel wall varied in different areas, an average of the maximum and minimum thicknesses was calculated.

##### 2.2.2.2 Assessment of ultrasonographic intestinal mucosal echogenicity changes caused by corn oil ingestion

The intestinal mucosa affected by corn oil ingestion was evaluated for changes in echogenicity. The changes were categorized as normal (nearly anechoic) or hyperechoic with different patterns observed, including uniformly hyperechoic mucosa, small nonlinear bright speckles, horizontal hyperechoic lines parallel to the submucosa, or vertical linear striations. The pattern of echogenicity change for each bowel segment at each time point was scored according to the specified criteria (Table 1). The scores were used to categorize the physiological activity of the intestinal mucosa as focal change, mild change in multiple segments, or diffuse change. The distribution of the patterned small intestine was categorized as focal, multi-segmental, or diffuse. The length and extent of the involved bowel segments were estimated based on longitudinal measurements. The presence or absence of mucosal echogenicity changes was recorded.

**Table 1:** Definition of the small bowel mucosal ultrasonographic echogenicity score.

Score	Description
1	Anechoic mucosa
2	Focal-sparse hyperechoic changes
3	Focal-moderate concentration of hyperechoic changes
4	Focal-abundant hyperechoic changes
5	Multi-segmental-sparse hyperechoic changes
6	Multi-segmental-moderate concentration of hyperechoic changes
7	Multi-segmental-abundant hyperechoic changes
8	Diffuse-sparse hyperechoic changes
9	Diffuse-moderate concentration of hyperechoic changes
10	Diffuse-abundant hyperechoic changes

### 2.3 Endoscopy

#### 2.3.1 Conventional endoscopic instrument and standard procedure

For endoscopy, dogs were prepared by fasting for at least 15 h. Prior to lower intestine endoscopy, the colon was thoroughly cleansed by performing two warm saline enemas. The first endoscopic examination was conducted in a fasted state 1 week after the ultrasound examination. Subsequent endoscopic procedures were performed at weekly intervals after oil ingestion at 3 and 12 h to allow for the recovery of the intestinal mucosa.

Etomidate (1 mg/kg) was administered as a pre-anesthetic drug followed by general anesthesia induced with alfaxane (2.2 mg/kg) and maintained with isoflurane. The dogs were intubated with a cuffed endotracheal tube, and the anesthesia level was regulated with isoflurane (0.5–3%).

Gastroduodenal endoscopy was performed using a flexible video endoscope (GIF-160, Olympus, Japan) with a working length of 110 cm, an outer diameter of 8.6 mm, and a working channel diameter of 2.8 mm. The dogs were positioned in the left lateral recumbent position, and the endoscope was inserted through the esophagus into the duodenum, passing through the pylorus. For lower intestinal endoscopy, the process started from the anus and progressed through the ileocolic valve into the ileum. Insufflation was used to maintain a clear view of the small intestine lumen as the endoscope was advanced. The depth of small intestinal access was estimated based on the centimeter markers on the endoscope. On average, a 30-cm section of the proximal duodenum and a 10-cm section of the terminal ileum were examined, but the endoscope could be advanced further if no resistance was encountered. Biopsy samples were obtained from normal-appearing duodenal and ileal mucosa using an Olympus biopsy forceps.

#### 2.3.2 Criteria for conventional endoscopic evaluation of small intestine mucosa

Mucosal change scores from conventional video recordings were reviewed for each dog. The mucosa of the duodenum and ileum were evaluated separately based on the upper and lower endoscopic procedures, respectively.

##### 2.3.2.1 Endoscopic gross findings of small intestinal mucosa

The quality of mucosal images was assessed for abnormalities such as foreign bodies, masses, polyps, or parasites. Various factors were evaluated to detect abnormalities, including inflation, hyperemia, vascularity, edema, discoloration, friability, texture, hemorrhage, erosion or ulceration, lacteal dilation, and the presence of mucus, bile, or food contents.

##### 2.3.2.2 Assessment of endoscopic intestinal mucosal changes caused by corn oil ingestion

A grading scale for mucosal physiological changes was established based on the appearance of villous edema, scattered pinpoint white foci, diffuse prominent mucosal roughness or granularity, or active lymphatic discharge (Table 2) (9). The grading scale criteria, such as villous parameter, extent, and descriptor, were used to determine mucosal change scores. The appearance of villous changes, their distribution (single, patchy, or diffuse), and extent of distribution (short segment, long segment, or whole segment) were recorded. The total score for each image was calculated based on the formula (Villous parameter × extent × descriptor) for the duodenum or ileum.

**Table 2:** Parameters and weights for endoscopy and capsule endoscopy scoring index^9^.

Parameters	Number	Longitudinal extent	Descriptors
Villous appearance	Normal – 0	Short segment (< 10%) – 8	Single – 1
	Edematous – 1	Long segment (11–50%) – 12	Patchy – 14
	Pinpoint white foci – 1	Whole segment (> 50%) – 20	Diffuse – 17

The grading scale for mucosal physiological changes was established based on villous appearance, scattered pinpoint white foci, diffuse prominent mucosal roughness or granularity, or active lymphatic discharge. For each image classified as duodenum, jejunum or ileum, a total score was computed with the formula (Villous parameter × extent × descriptor).

### 2.4 Capsule endoscopy

#### 2.4.1 Capsule endoscopic instrument and standard procedure

The first capsule endoscopic examination was conducted 1 week after the completion of conventional endoscopic examination. Dogs were fasted for 15 h with access to water before the procedure. Enema and anesthesia were not required. Subsequent capsule endoscopic procedures were performed at weekly intervals to prevent the accumulation of previous effects of the oil and increase the fasting interval.

The MiroCam® Capsule Endoscope System (MC1200-M, IntroMedic Inc., Seoul, Republic of Korea) was used in the study. The capsule, measuring 24.5 × 10.8 mm (length × diameter), contained a charge-coupled device camera, light emitting diodes, a battery, and a radiofrequency transmitter. It had a 170° field of view and captured images at a rate of six frames per second. The capsule’s battery life allowed data to be received by external antennae and saved in the receiver MR2000 unit for approximately 12 h. Data were later uploaded to a workstation for analysis. Real-time viewing of the transmitted capsule images was possible using the receiver MR2000 unit.

Dogs were fitted with a full-body stockinette with slits in the chest and abdominal regions for the antennae. The receiver unit was placed in a pocket on the stockinette, and an Elizabethan collar was used to prevent the dog from chewing the instrument. The capsule endoscope was administered orally with 5 mL of water, and the dog was allowed to resume its daily activities. The progress of the capsule along the GI tract was periodically monitored using a real-time viewer and endoscopic monitoring. Data were analyzed using the MiroView 4.0 software.

#### 2.4.2 Criteria of capsule endoscopic small intestine mucosa evaluation

The transit time between the first duodenal and cecal images captured by the capsule was divided into three equal intervals, with each interval individually scored. The extent of mucosal change in each tertile was determined by assessing the percentage of a particular tertile showing mucosal change. A short segment was defined as <10% of a tertile, a long segment as 11–50% of a tertile, and a whole segment as >50% of a tertile (9). At least 150 consecutive frames (25 s) in each segment were analyzed, and the total score was calculated based on the villous parameter, extent, and descriptor.

The cleanliness of the images was graded for the presence of bubbles and dark or opaque small bowel contents. The series of images in each tertile were assigned a total score using the formula described in Table 2. The maximum tertile score, calculated as villous parameter × extent × descriptor, was used as the total score for each segment (9).

### 2.5 Histopathological evaluation

Histopathological evaluation involved processing the biopsy samples from the duodenum and ileum. Samples were fixed in 10% neutral-buffered formalin and processed through dehydration and clearing. Samples were then embedded in paraffin wax and sectioned into 4-μm sections to expose the long axis of the villi. Hematoxylin and eosin staining was performed on the sections to visualize the tissue.

Before evaluating the biopsy samples, high-quality slides with sufficient samples were selected for each time point (fasting, and 3 h and 6 h after oil ingestion) and intestinal site. The quality of the histological samples was assessed following the World Small Animal Veterinary Association (WSAVA) guidelines (10) to grade the local villus architecture (depth), determine the number of dilated lacteals present, the degree of dilation, and the mucosal involvement, including submucosal changes. The severity of the lacteal dilation was assigned as within physiological change if the width of the central lacteal, as a percentage of the width of the lamina propria, was more than 25%. Histopathological evaluation was used to determine whether the samples showed normal change, using as criteria the quality of the histological samples, the lacteal dilation in the villi, and the absence of histological abnormalities in the samples.

A criterion for the histological diagnosis of the physiological change consisting of intestinal lymph vessel dilation was established, namely the number of villi in which the dilation of the lymphatic vessels was confirmed, even if mild, divided by the total number of villi observed on the slide. This criterion was chosen to alleviate the effects of the assessment of the relative severity of the lacteal dilation. To examine the degree of physiological lacteal dilation, all samples were graded and scored under 4× magnification.

### 2.6 Statistical analysis

Statistical analysis was performed using the SPSS 25.0 program. Data were expressed as mean ± standard deviation. Repeated measures analysis of variance (ANOVA) was used to assess differences between the evaluation scores of the duodenum, jejunum, and ileum and the percentage of dilated villi. Bonferroni post-hoc t-test was applied to assess differences in scores between the duodenal and ileal regions based on the test method. A *p*-value of <0.05 was considered statistically significant in all analyses.

## 3 Results

### 3.1 Comparison of ultrasonographic mucosal echogenicity changes over time

**Figure 1:**
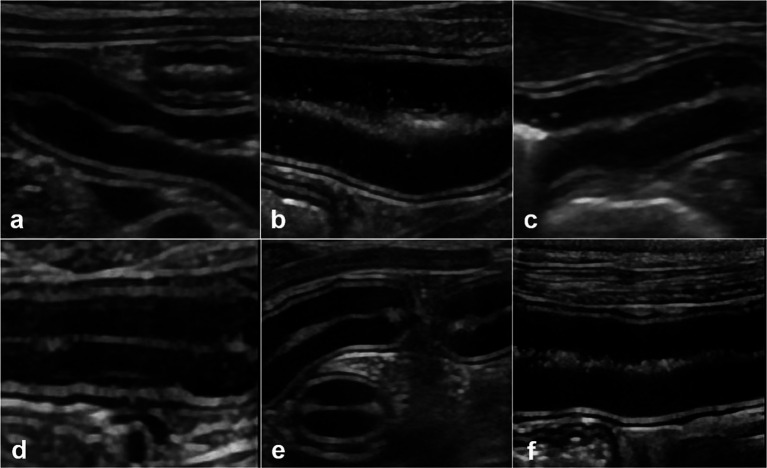
Ultrasonographic small intestinal mucosal echogenicity changes in dog 1. This figure represents six frames of ultrasonographic mucosa at different times. The entire small intestine of dog 1 is scanned in real time and mucosal echogenicity scores are evaluated at fasting **(A)**, and at 0 h **(B)**, 1 h **(C)**, 3 h **(D)**, 6 h **(E)**, and 12 h **(F)** after oil ingestion. The conspicuity of hyperechoic foci **(B,C)**, or horizontal lines parallel to the submucosa **(D)**, in one or more segments of the duodenum, jejunum, or ileum is observed as early as immediately after ingestion and remained prominent up to 3 h later **(B–D)**.

Ultrasound and endoscopic images were reviewed in a blinded manner, without the reviewer being aware of the specific time points. Ultrasonographic evaluation of mucosal echogenicity changes showed that there were no pathological conditions observed in the small intestine of the dogs at any time point (Figure 1). Wall thicknesses of the entire small intestine did not exceed the upper normal limits, indicating normal physiological changes rather than pathological ones (Table 3).

**Table 3:** Wall thickness of duodenum, jejunum, and ileum measured using ultrasound as a function of the time elapsed after ingestion of corn oil.

	Mean ± SD (mm)						*p*
	Fasting	0 h	1 h	3 h	6 h	12 h	
Duodenum	4.14 ± 0.85	4.43 ± 0.61	4.44 ± 0.55	4.28 ± 0.66	4.53 ± 0.57	4.37 ± 0.62	0.067
Jejunum	3.25 ± 0.51	3.15 ± 0.67	3.38 ± 0.53	3.38 ± 0.62	3.43 ± 0.58	3.23 ± 0.58	0.081
Ileum	2.67 ± 0.65	2.80 ± 0.69	3.00 ± 0.69	2.89 ± 0.96	2.76 ± 0.81	2.93 ± 0.86	0.405

Changes in duodenum, jejunum, and ileum wall thickness at fasting, and at 0 h, 1 h, 3 h, 6 h, and 12 h post corn oil administration are shown (mean ± SD of all experimental dogs). The wall thickness for the entire small intestine is not significantly different between the experimental dogs before or at any point after treatment.

After ingesting corn oil, all dogs showed a temporary increase in mucosal echogenicity and the presence of hyperechoic foci or horizontal lines parallel to the submucosa in one or more segments of the duodenum, jejunum, or ileum. Representative images displayed the progression of these changes in the small intestine after corn oil ingestion.

Mucosal echogenicity scores for each intestinal segment at each time point were evaluated (Table 4). Mucosal echogenicity scores gradually returned to fasting levels 12 h after ingesting corn oil. The scores were focal or multisegmental, with some segments exhibiting higher scores than others. The highest score assigned to each dog was for any duodenum segment between 0 and 3 h after oil ingestion.

**Table 4:** Ultrasonographic echogenicity scores of duodenum, jejunum, and ileum mucosa as function of the time elapsed after ingestion of corn oil.

	Mean ± SD					
	Fasting	0 h	1 h	3 h	6 h	12 h
Duodenum	2.40 ± 0.55	6.60 ± 2.51	4.80 ± 2.28	4.60 ± 3.05	2.80 ± 1.64	2.40 ± 0.89
Jejunum	2.40 ± 0.55	6.00 ± 1.22*	2.60 ± 1.34	3.40 ± 1.52	2.40 ± 0.55	2.20 ± 0.84
Ileum	1.20 ± 0.45	1.80 ± 0.84	2.80 ± 0.45†	3.20 ± 1.79	2.20 ± 1.64	1.60 ± 0.89

Ultrasonographic echogenicity scores for each intestinal segment at each time point (mean ± SD of all experimental dogs). Mucosal echogenicity scores in the whole intestine recover to fasting levels 12 h after ingesting corn oil. All other time points are evaluated based on the increase in mucosal echogenicity compared with fasting levels. The duodenal echogenicity score is not significantly different between time points. The jejunum shows a significantly greater echogenicity score 0 h after oil ingestion compared with fasting level (*p < 0.05). The ileum shows significantly greater echogenicity score at the 1 h time point compared with fasting level (†*p* < 0.05), and the other time points are not significantly different.

Statistical analysis revealed that the duodenal echogenicity score was not significantly different between time points but was lowest at fasting, highest at 0 h, and restored to fasting levels at later time points. The jejunum showed a significantly greater echogenicity score at 0 h than during fasting. The ileum had a significantly greater echogenicity score at 1 h than during fasting, while the other time points did not show significant differences.

The increased mucosal echogenicity and visibility of lacteals and lymphatic vessels observed in images were attributed to the uptake of fat from lacteals, which is a normal physiological process. The presence of speckles and hyperechoic parallel lines within the mucosa was considered a normal finding and not indicative of a pathology. No hyperechoic mucosal vertical striations were observed at any time point.

Overall, ultrasonographic evaluation demonstrated temporary changes in mucosal echogenicity after corn oil ingestion, with the mucosa gradually returning to that during fasting. These changes were considered physiological rather than pathological.

### 3.2 Comparison of endoscopic gross mucosal changes over time

Endoscopic images were reviewed in a blinded manner, without the reviewer being aware of the specific time points. Endoscopic examination of the small intestinal mucosa showed that the typical appearance of the mucosa with digitate villi was observed in all dogs at different time points. The color of the mucosa varied within the normal range of pinkish white, reddish pink, yellow red, or light red. No significant abnormal characteristics were observed, and the detected changes were within the normal variation.

**Figure 2:**
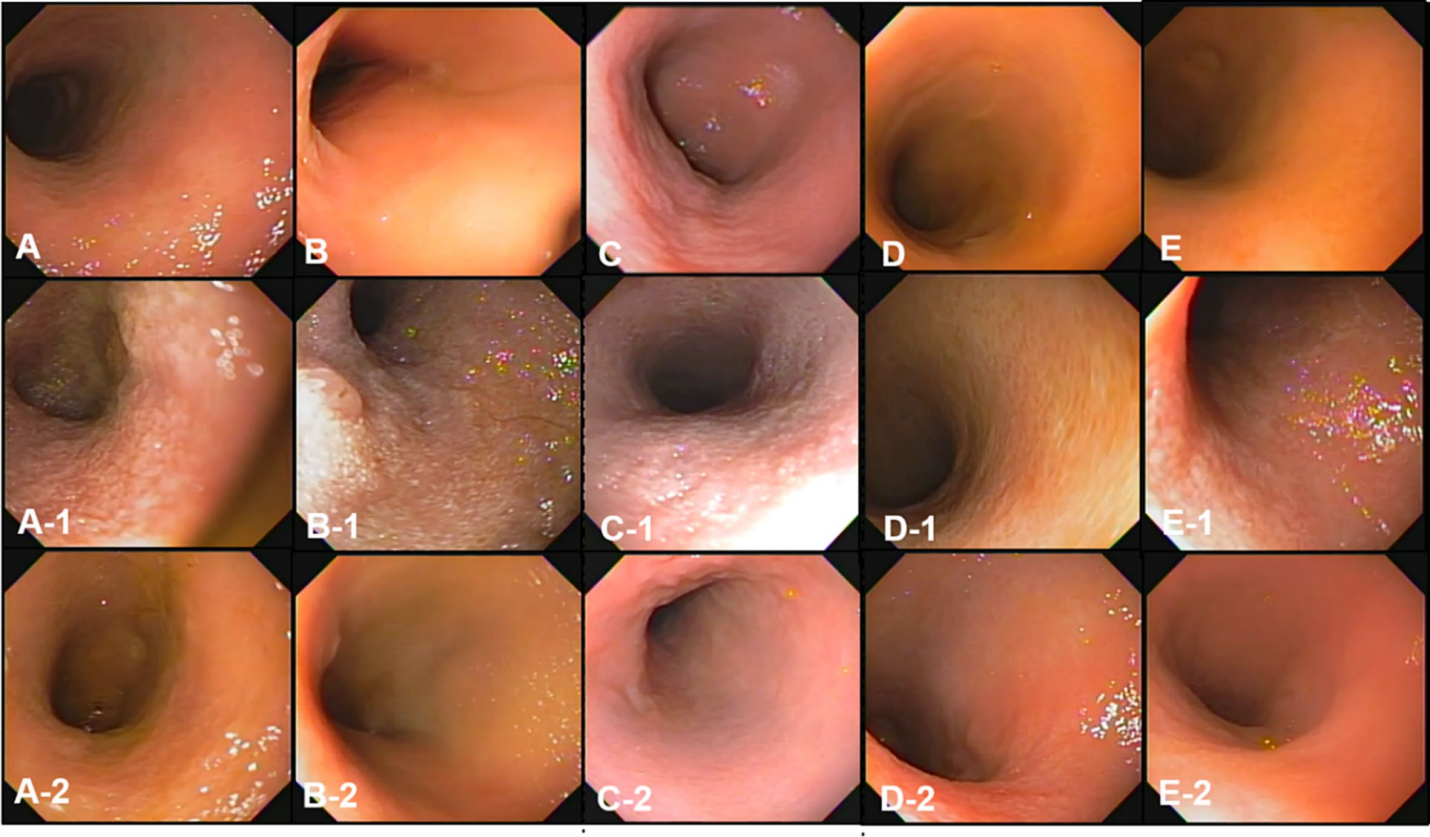
The gross mucosal appearance of duodenum conventional endoscopy at fasting **(A–E)** and 3 h **(A-1,B-1,C-1,D-1,E-1)** and 12 h **(A-2,B-2,C-2,D-2,E-2)** after oil ingestion. All dogs show normal mucosal appearance and the color is pinkish white to yellowish red. The mucosal roughness, pinpoint whitish mucosal foci and diffuse edematous aspect at 3 h **(A-1,B-1,C-1)** are somewhat confirmed, compared with the images at fasting (**A,A-1,A-2**; Dog 1/**B,B-1,B-2**; Dog 2/**C,C-1,C-2**; Dog 3/**D,D-1,D-2**; Dog 4/**E,E-1,E-2**; Dog 5).

**Figure 3:**
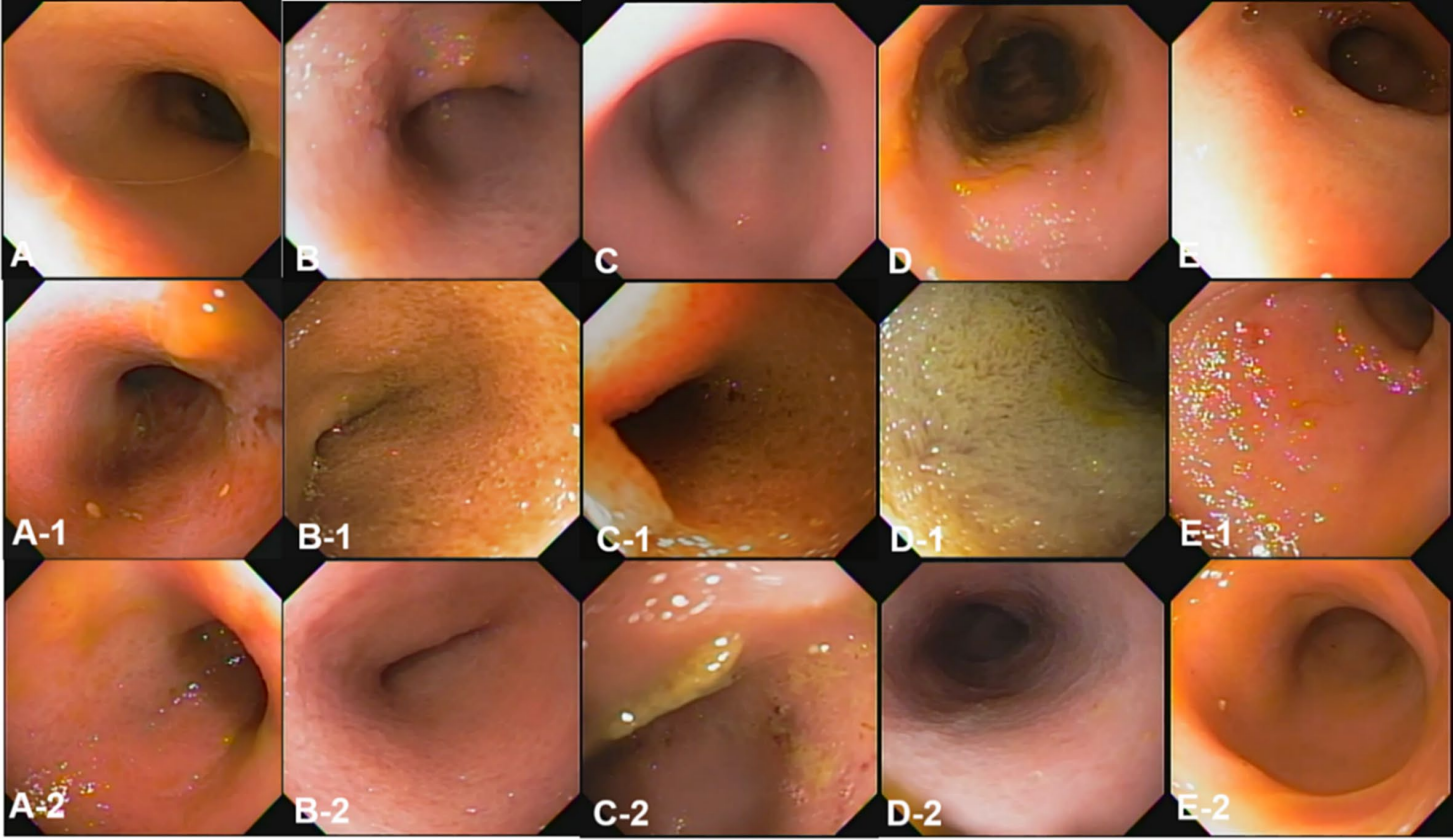
The gross mucosal appearance of the ileum on conventional endoscopy at fasting **(A–E)** and 3 h **(A-1,B-1,C-1,D-1,E-1)** and 1 2 h **(A-2,B-2,C-2,D-2,E-2)** after oil ingestion. All dogs show normal mucosal appearance and the color is pinkish white to yellowish red. The mucosal roughness, pinpoint whitish mucosal foci and diffuse edematous villi of the ileum at 3 h **(A-1,B-1,C-1)** are somewhat confirmed, compared with images at fasting (**A,A-1,A-2**; Dog 1/**B,B-1,B-2**; Dog 2/**C,C-1,C-2**; Dog 3/**D,D-1,D-2**; Dog 4/**E,E-1,E-2**; Dog 5).

At pre-treatment (fasting), 3 h, and 12 h after oil ingestion, the mucosal roughness and diffuse edematous aspect were somewhat confirmed compared to the fasting images (Figures 2, 3). Pinpoint whitish mucosal foci were also observed in some dogs, indicating significant changes. The edematous villi of the ileum showed remarkable changes at 3 h after oil ingestion.

The conventional endoscopic evaluation scores for fasting, 3 h, and 12 h after oil ingestion were compared statistically at different time points using repeated measures ANOVA. Despite the evident changes observed before and after corn oil ingestion, there were no significant differences among the conventional endoscopic evaluation scores (Table 5).

**Table 5:** Conventional endoscopic evaluation scores in the duodenum and ileum at fasting and 3 h and 12 h after oil ingestion.

	Mean ± SD		
	Fasting	3 h	12 h
Duodenum	7.00 ± 1.87	32.00 ± 17.66	10.80 ± 4.02
Ileum	5.25 ± 3.30	12.75 ± 6.18	4.50 ± 1.29

Scores at fasting, and 3 h and 12 h after oil ingestion (mean ± SD of all experimental dogs). The conventional endoscopic evaluation scores are not significantly different in the all experimental dogs before or at any point after treatment.

Overall, endoscopic evaluation of the mucosal changes did not reveal significant differences in the conventional endoscopic evaluation scores before and after corn oil ingestion.

The observed changes were within the normal variation and did not indicate any pathological conditions.

### 3.3 Comparison of capsule endoscopic mucosal changes over time

**Figure 4:**
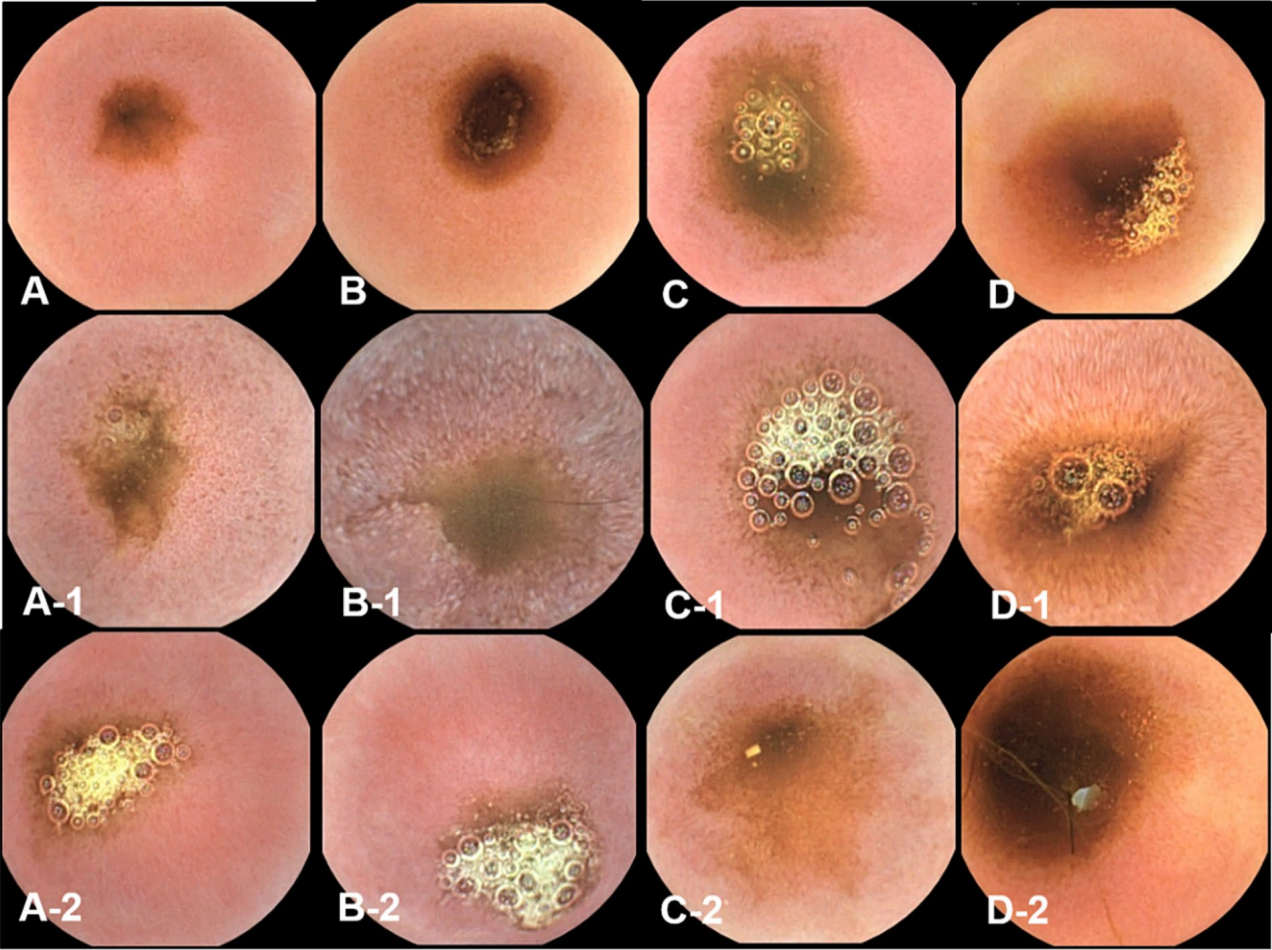
The gross mucosal appearance of duodenum capsule endoscopy at fasting **(A–D)** and 3 h **(A-1,B-1,C-1,D-1)** and 12 h **(A-2,B-2,C-2,D-2)** after oil ingestion. In comparison with fasting capsule endoscopic images, at 3 h, the diffuse edematous aspect of mucosa is clearly observed, especially in the duodenum and jejunum **(B-1,D-1)**. Pinpoint whitish foci are also seen in the duodenum (Figure 8B–1) (**A,A-1,A-2**; Dog 1/**B,B-1,B-2**; Dog 2/**C,C-1,C-2**; Dog 3/**D,D-1,D-2**; Dog 4).

**Figure 5:**
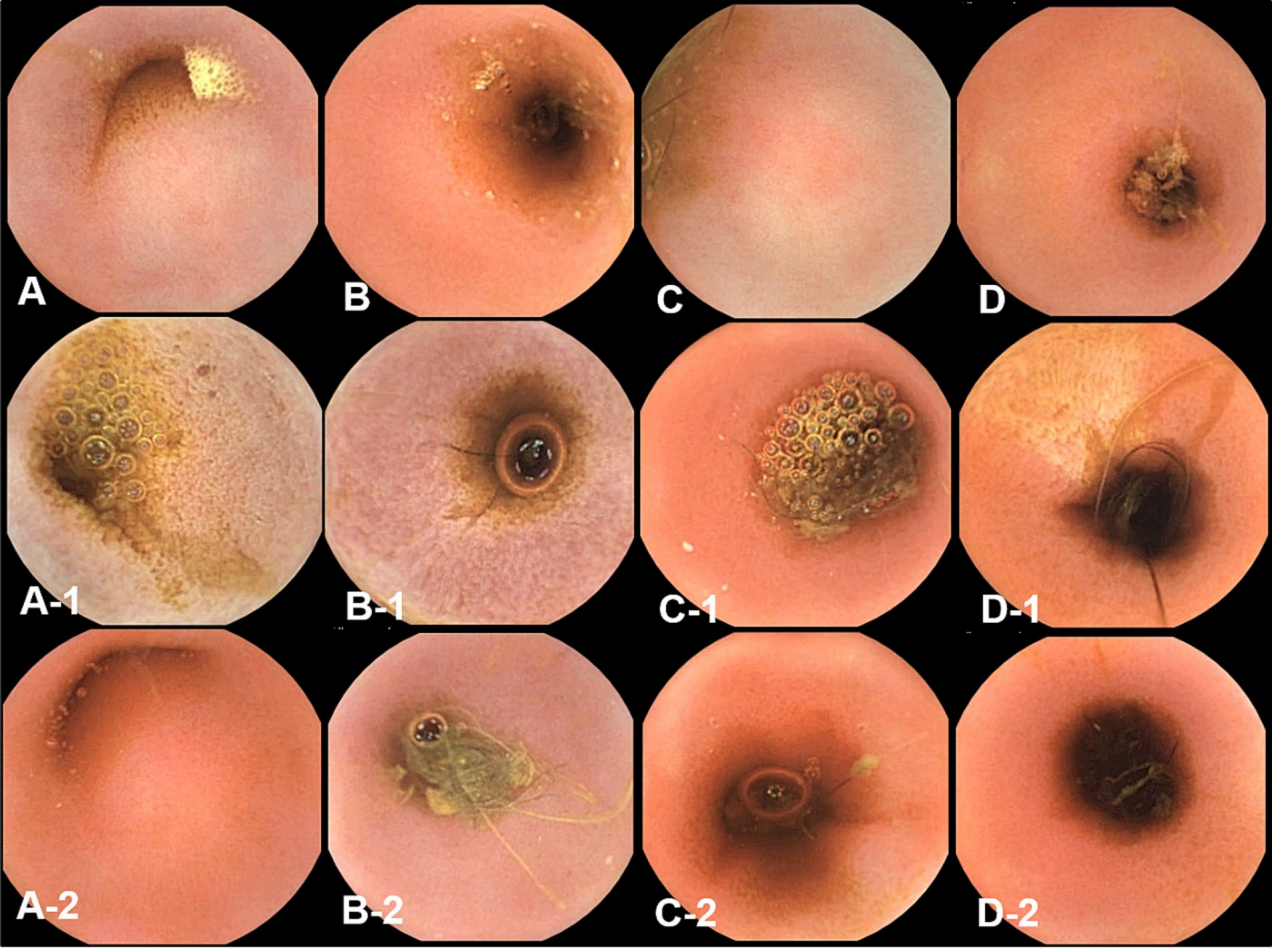
The gross mucosal appearance of jejunum capsule endoscopy at fasting **(A–D)** and 3 h **(A-1,B-1,C-1,D-1)** and 12 h **(A-2,B-2,C-2,D-2)** after oil ingestion. In comparison with fasting capsule endoscopic images, at 3 h, the diffuse edematous aspect of mucosa is clearly observed, especially in the duodenum and jejunum **(A-1,B-1)** (**A,A-1,A-2**; Dog 1/**B,B-1,B-2**; Dog 2/**C,C-1,C-2**; Dog 3/**D,D-1,D-2**; Dog 4).

**Figure 6:**
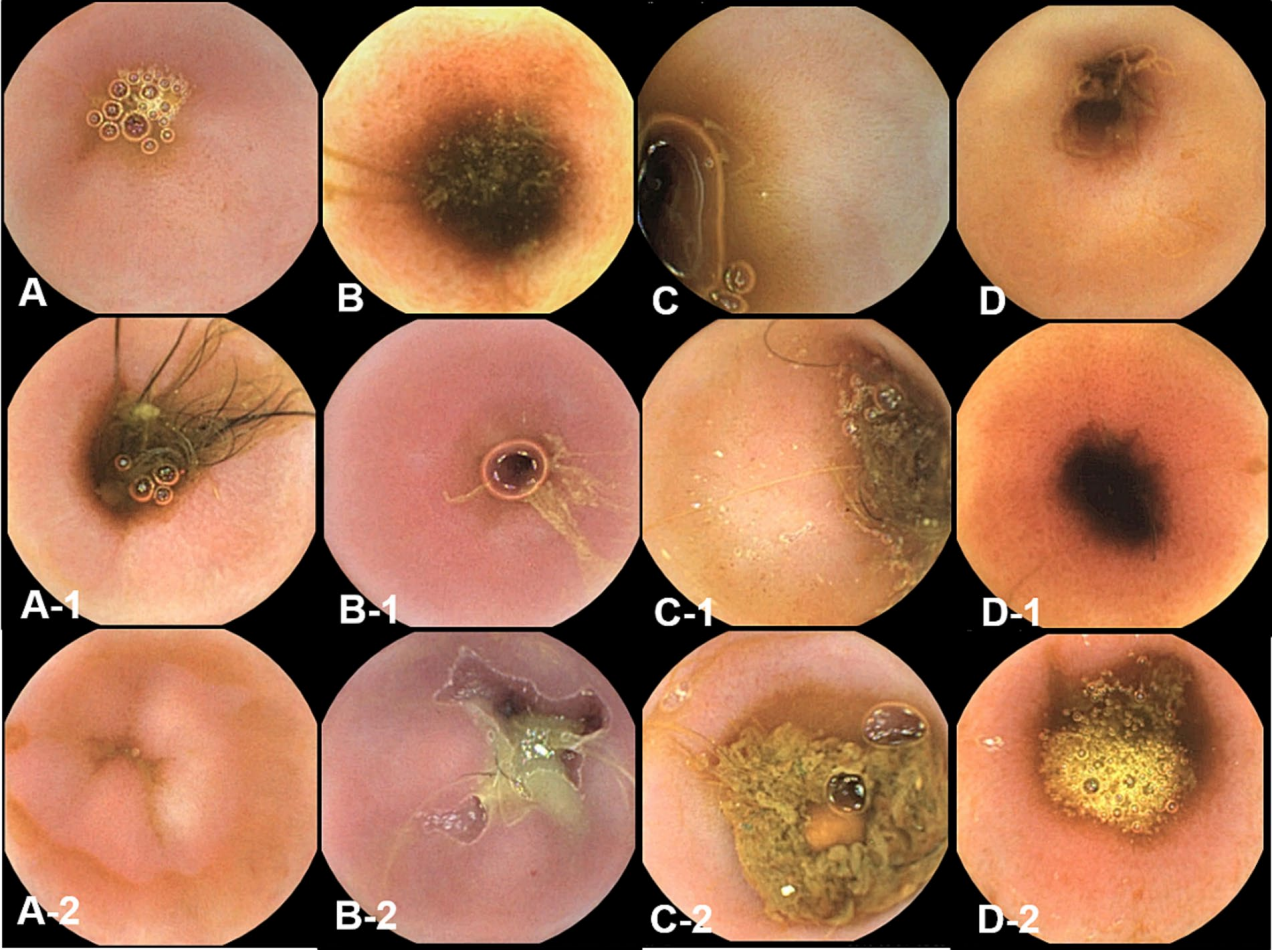
The gross mucosal appearance of ileum capsule endoscopy at fasting **(A–D)** and 3 h **(A-1,B-1,C-1,D-1)** and 12 h **(A-2,B-2,C-2,D-2)** after oil ingestion. In comparison with fasting capsule endoscopic images, no significant changes in ileum are found (**A,A-1,A-2**; Dog 1/**B,B-1,B-2**; Dog 2/**C,C-1,C-2**; Dog 3/**D,D-1,D-2**; Dog 4).

Capsule endoscopy was used to evaluate the mucous membrane of entire small intestine including duodenum, jejunum, and ileum overcoming the limitation of endoscopic tubes (Figures 4–6).

Capsule endoscopic images were reviewed in a blinded manner, without the reviewer being aware of the specific time points.

Due to a prolonged gastric transit time in dog 5, no images were obtained as the capsule remained in the stomach beyond the battery life of the endoscope. In contrast, the gastric transit time in the remaining four dogs during the capsule endoscopy examination ranged from 2 to 3 h, with the capsule endoscope passing through the entire small intestine in less than 2 h. There was no significant difference in gastric transit time before and after the administration of oil.

Comparison of capsule endoscopic images with fasting images revealed diffuse edematous aspects of the mucosal membrane, especially in the duodenum and jejunum, at 3 h after corn oil ingestion. Pinpoint whitish foci were also seen in the duodenum at 3 h.

Repeated measures ANOVA was performed to assess the differences in the evaluation scores of the duodenum, jejunum, and ileum over time (Table 6). Duodenal and jejunal evaluation scores showed significant differences between time points. Specifically, the duodenal mucosal change was higher at 3 h than during fasting. The jejunal capsule endoscopic scores also differed significantly between time points, with the highest score observed at 3 h compared to that at 12 h and during fasting. No significant differences were found in the ileum scores.

**Table 6:** Capsule endoscopy evaluation scores in the duodenum, jejunum, and ileum at fasting and 3 h and 12 h after oil ingestion.

	Mean ± SD		
	Fasting	3 h	12 h
Duodenum	9.75 ± 3.30	45.00 ± 10.00*	22.00 ± 6.78
Jejunum	6.75 ± 3.30	25.50 ± 9.00†	11.00 ± 5.23
Ileum	5.60 ± 0.55	23.80 ± 16.44	9.80 ± 1.30

Changes of capsule endoscopic mucosa evaluation score of duodenum, jejunum, and ileum as a function of time after ingestion of corn oil (mean ± SD of all experimental dogs). The duodenal and jejunum evaluation scores differ significantly among time points. Specifically, the duodenal mucosal change is observed to be higher at 3 h compared with fasting (*p < 0.05). The jejunum capsule endoscopic scores also differ significantly among time points, and the score is higher at 3 h compared with 12 h and fasting (†*p* < 0.05). No significant differences in ileum scores are found.

Overall, capsule endoscopic evaluation demonstrated significant mucosal changes in the duodenum and jejunum at 3 h after corn oil ingestion. These changes included diffuse edematous aspects and pinpoint whitish foci. However, no significant changes were observed in the ileum.

### 3.4 Comparison of the percentage of dilated villi over time on histopathological evaluation

**Figure 7:**
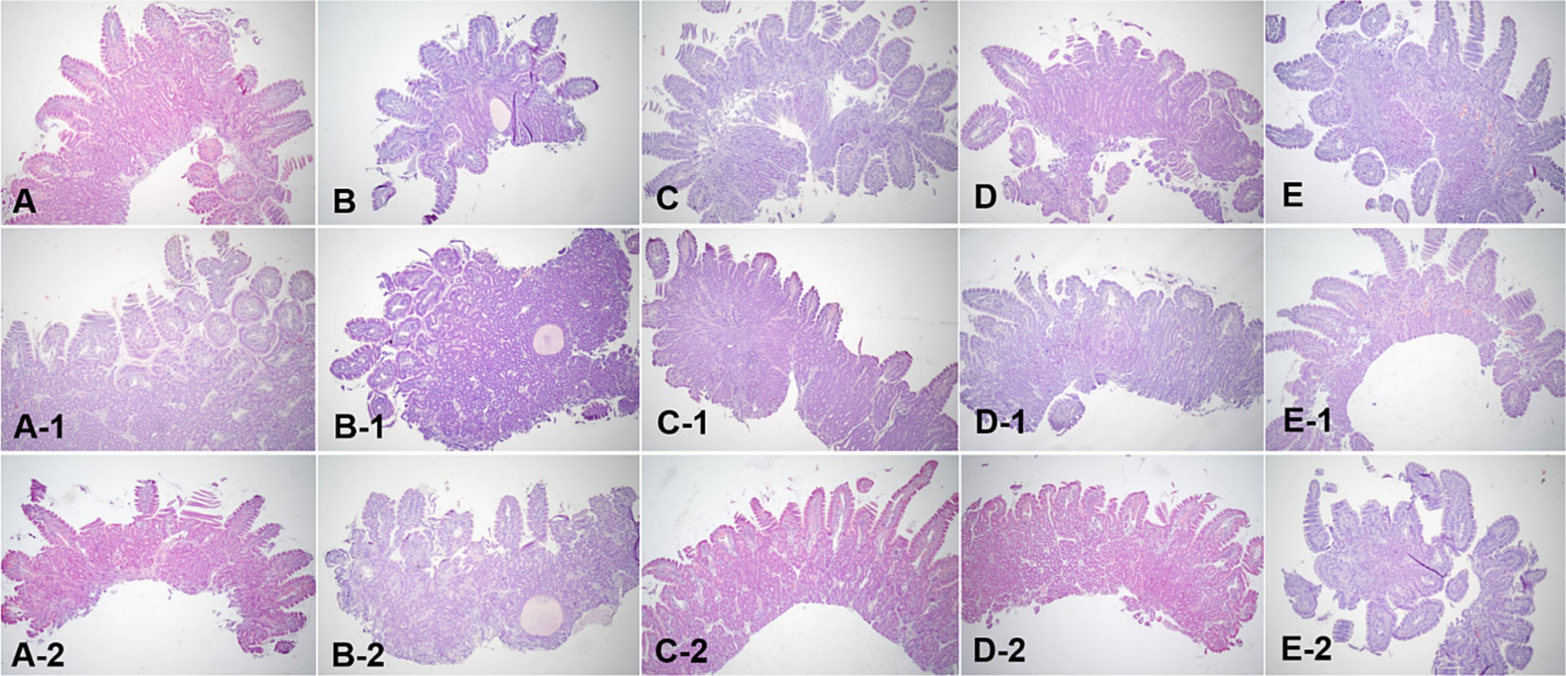
Microscopic examination of the duodenum villi biopsy samples at fasting **(A–E)**, and 3 h **(A-1,B-1,C-1,D-1, E-1)** and 12 h **(A-2,B-2,C-2,D-2,E-2)** after oil ingestion. Variable degrees of lacteal dilation, within normal variation, are present in these tissues. Normal architecture of small intestinal mucosa is apparent, and the percentage of dilated lacteals is not significant (**A,A-1,A-2**; Dog 1/**B,B-1,B-2**; Dog 2/**C,C-1,C-2**; Dog 3/**D,D-1,D-2**; Dog 4/**E,E-1,E-2**; Dog 5). H&E stains. Original magnification: 40 ×.

**Figure 8:**
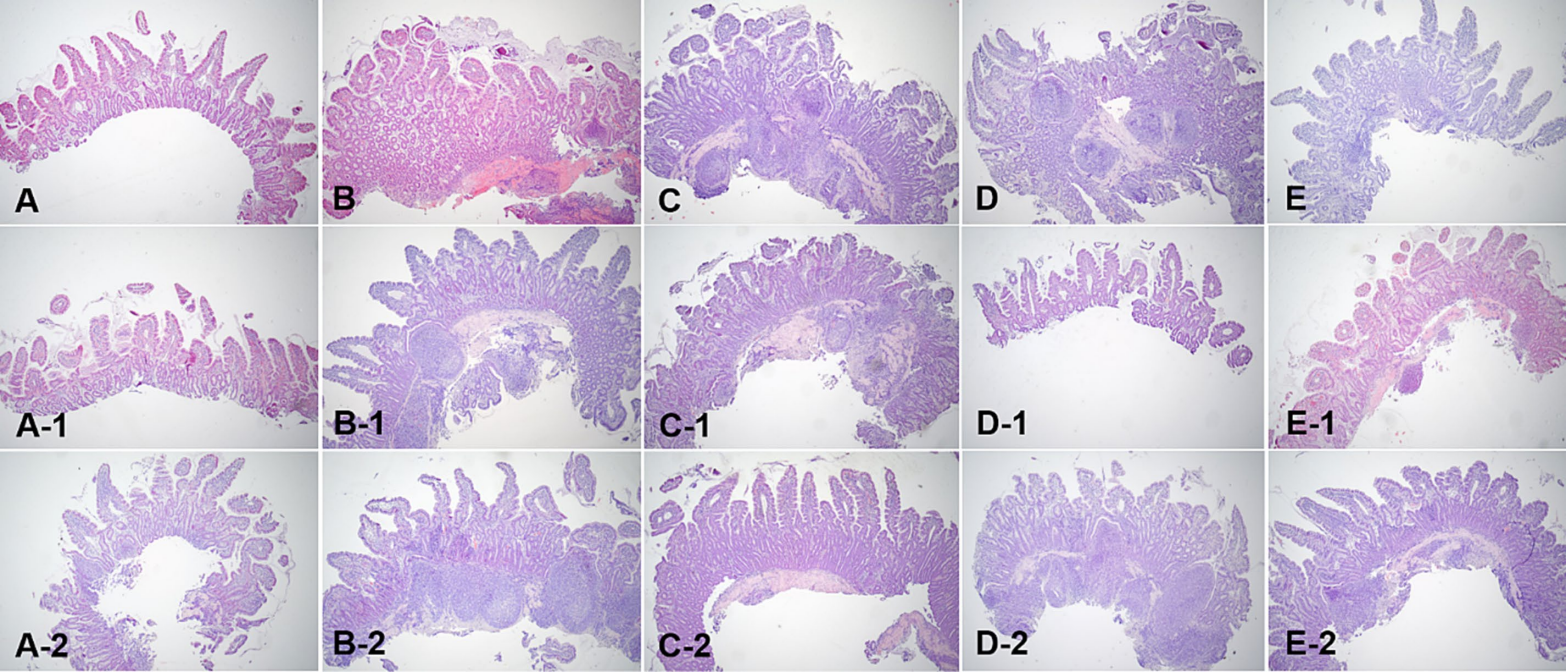
Microscopic examination of ileum villi biopsy samples at fasting **(A–E)**, and 3 h **(A-1,B-1,C-1,D-1, E-1)** and 12 h **(A-2,B-2,C-2,D-2,E-2)** after oil ingestion. Variable degrees of lacteal dilation, within normal variation, are present in these tissues. The ratio of the dilated villi to total villi in ileum is higher at 3 h than at 12 h (**A,A-1,A-2**; Dog 1/**B,B-1,B-2**; Dog 2/**C,C-1,C-2**; Dog 3/**D,D-1,D-2**; Dog 4/**E,E-1,E-2**; Dog 5). H&E stains. Original magnification: 40 ×.

Histopathological evaluations were reviewed in a blinded manner, without the reviewer being aware of the specific time points. Histopathological evaluation of the duodenal and ileal biopsy specimens obtained from the dogs using conventional endoscopy showed that all intestinal mucosal tissues were normal. The tissue quality of the samples ranged from marginal to adequate, and appropriate sections were obtained to observe the long axis of the villi in cross-section (Figures 7, 8).

Variable degrees of lacteal dilatation, all within normal variation, were present in the dogs. The percentage of dilated lacteals in the duodenum and ileum is presented in Table 7. Repeated measures ANOVA was performed to assess the ratio of dilated villi to total villi in the duodenum and ileum over time.

**Table 7:** Percentage of dilated villi of duodenum and ileum at fasting, and 3 h and 12 h after oil ingestion.

	Mean ± SD		
	0 h	3 h	12 h
Duodenum	47.66 ± 4.62	58.38 ± 5.41	48.58 ± 2.88
Ileum	38.40 ± 10.20	53.00 ± 12.55*	47.36 ± 12.37

Percentages of dilated lacteals (mean ± SD of all experimental dogs). Differences in the duodenum are not significant, but they are in the ileum: specifically, the percentage of dilated ileum villi is higher at 3 h than at 12 h (*p < 0.05).

The differences in the duodenum were not significant, indicating that the percentage of dilated villi did not vary significantly over time in this region. However, in the ileum, the percentage of dilated villi was significantly higher at 3 h than at 12 h.

These findings suggest that there is an increase in the percentage of dilated villi in the ileum at 3 h after corn oil ingestion, which subsequently decreases by the 12-h time point. This indicates a transient effect of corn oil ingestion on the dilation of ileum villi.

## 4 Discussion

The diagnosis of IL relies on the histopathological identification of dilated lacteals containing proteinaceous material. The severity of lymphatic dilation is assessed based on the number of dilated lacteals, the degree of dilation, and the location of dilation within the intestinal layers (3). In this study, physiological lymphatic dilation within the normal range was observed, indicating functional lymphangiectasia. Previous studies have reported transient lymphangiectasia because of fat transport disturbances (3, 7). Similarly, in this study, the ingestion of high-fat oil likely caused a transient disturbance of fat transport, leading to physiological lymphatic dilation.

Ultrasound examination evaluated changes in the intestinal mucosa after high-fat ingestion. LCTs are mainly transported into intestinal lacteals in the distal duodenum and jejunum whereas medium-chain triglycerides are absorbed directly into the portal venous system (11–13). Ultrasonography revealed hyperechoic speckles and horizontal stripes within the mucosal layer, which are indicative of physiologic lacteal dilation. These findings align with those of previous studies and suggest that the observed changes are not pathological (4, 5, 8). The absence of vertical striations, typically associated with lacteal dilation, further supports the notion that the changes observed were within the physiological range (14, 15). From the ultrasonographic evaluation, results demonstrated that corn oil ingestion triggered a temporary increase in mucosal echogenicity in the duodenum, jejunum, and ileum. This transient rise in echogenicity reflected the physiological processes of fat uptake by lacteals and was not indicative of pathological changes. This aspect of the study provides valuable knowledge about normal physiological adaptations of the small intestine to fat ingestion.

Conventional endoscopy and capsule endoscopy were utilized to assess mucosal changes in the small intestine. Capsule endoscopy, with its ability to visualize the entire small bowel, provided valuable information regarding mucosal changes (16). The Lewis score, a standardized scoring system, was used to evaluate the severity of mucosal changes observed during capsule endoscopy (9).

According to our study results, conventional endoscopy showed no significant differences before or at any time point after treatment, while capsule endoscopy evaluation scores indicated a difference. Tables 5, 6 present the numerical results of conventional and capsule endoscopy, respectively. Upon review, both endoscopic methods show numerical changes following oil administration. However, for conventional endoscopy, no statistically significant difference was found, even though Table 5 reveals clear numerical differences. We have considered potential reasons for the statistical discrepancies between conventional and capsule endoscopy. One potential explanation is that during conventional endoscopy, air is continuously insufflated to distend the lumen, which may contribute to variations in the numerical evaluations. In contrast, capsule endoscopy moves through the gastrointestinal tract via natural peristalsis without inflating the mucosa, which may partly explain the differences in mucosal evaluation scores between the two methods. Another factor could be the difference in image quality and resolution between conventional and capsule endoscopy.

A previous study reported that feeding dogs a high-fat diet delayed gastric transit time during capsule endoscopy, resulting in prolonged gastric retention, and thus recommended avoiding a high-fat diet prior to the procedure (5). Fortunately, in our study, there was no observed difference in gastric transit time during capsule endoscopy following fat administration, likely due to the small amount of fat provided, which may have had minimal impact.

Histological examination of biopsy samples obtained during endoscopy confirmed that the changes in the mucosa were physiological. The correlation between endoscopic appearance and histological changes can be influenced by the quality of tissue samples obtained. Inadequate tissue samples may lead to erroneous diagnoses and lower sensitivity in identifying intestinal lesions (6, 10). Additionally, systemic symptoms of inflammation may not always be reflected in the endoscopic appearance of the mucosa, contributing to the limited correlation between the two (17). Histopathological evaluation furthered the understanding of the physiological impact of corn oil by showing an interesting phenomenon of temporary villi dilation, particularly noticeable in the ileum at 3 h after oil ingestion. This indicates a transient structural adaptation of the villi to accommodate increased fat absorption, thereafter reverting to the normal state after 12 h.

Altogether, this research reaffirms the resilience and adaptive nature of the small intestine to dietary changes, specifically corn oil ingestion, in healthy dogs. The findings shed light on the physiological changes that ensue following the ingestion of fats and further validate the use of ultrasonography, endoscopy, and capsule endoscopy as effective tools for visualizing these changes. However, as these changes were observed in healthy dogs, additional investigations in dogs with GI diseases are warranted to examine if similar physiological adaptations occur and to determine whether these changes could have any clinical significance.

It is important to note that this study was conducted on a small number of healthy dogs, and the results should be validated in a larger population, including dogs with enteropathy. Although the test results were reviewed in a blinded manner, the evaluations were conducted by a single examiner, and the subjective nature of quantifying physiological changes may introduce variability. The timing and sequence of different examinations were not simultaneous, which could potentially affect the results. Future studies should aim to address these limitations for a more comprehensive understanding of IL and its diagnostic modalities.

In conclusion, this study demonstrated that the observed physiological changes in the small intestinal mucosa, including lymphatic dilation, hyperechoic speckles, and stripes, were within the normal range after oil ingestion (0.5 mL/kg) in healthy Beagle dogs. The use of various endoscopic techniques provided valuable insights into the mucosal changes after oil ingestion. In particular, capsule endoscopy, which allows visual changes in the entire small intestine to be confirmed without anesthesia, was very useful in this experiment.

Mild mucosal changes were observed immediately after fat ingestion and persisted for up to 3 h. However, these changes gradually resolved by the 12-h mark, allowing for differentiation between normal and diseased intestines. This study may serve as a baseline for future research. Any changes that persist beyond 12 h may indicate pathological alterations and could potentially aid in the diagnosis of intestinal lymphangiectasia.

## Data Availability

The raw data supporting the conclusions of this article will be made available by the authors, without undue reservation.
